# Supplementary material for: HIVprotI: an integrated web based platform for prediction and design of HIV proteins inhibitors
Source: J Cheminform. 2018 Mar 9;10:12. doi: 10.1186/s13321-018-0266-y (PMC5845081; doi:10.1186/s13321-018-0266-y)
Supplement: Supplementary file 2 — Additional file 2. Source code of HIVProtI web server. [file 13321_2018_266_MOESM2_ESM.zip › HIVprotI_Source-code/hivprotI/weka-3-6-6/doc/weka/core/Copyable.html]

Copyable


---


|  |  |  |
| --- | --- | --- |
| **Overview** \| **Package** \| **Class** \| **Tree** \| **Deprecated** \| **Index** \| **Help** \| **Weka's home** | |  |
| **PREV CLASS**   **NEXT CLASS** | **FRAMES**    **NO FRAMES**     **All Classes** |  |
| SUMMARY: NESTED \| FIELD \| CONSTR \| METHOD | DETAIL: FIELD \| CONSTR \| METHOD |  |


---


## weka.core Interface Copyable

**All Known Implementing Classes:**: Attribute, BestFirst.LinkedList2, BinarySparseInstance, FastVector, IndividualInstance, Instance, LFSMethods.LinkedList2, Rule, SparseInstance

---

``` public interface Copyable ```

Interface implemented by classes that can produce "shallow" copies
of their objects. (As opposed to clone(), which is supposed to
produce a "deep" copy.)

**Version:**
:   $Revision: 1.6 $

**Author:**
:   Eibe Frank (eibe@cs.waikato.ac.nz)

---

| **Method Summary** | |
| --- | --- |
| `java.lang.Object` | `copy()`             This method produces a shallow copy of an object. |

**Method Detail**

### copy

```
java.lang.Object copy()
```

:   This method produces a shallow copy of an object.
    It does the same as the clone() method in Object, which also produces
    a shallow copy.


---


|  |  |  |
| --- | --- | --- |
| **Overview** \| **Package** \| **Class** \| **Tree** \| **Deprecated** \| **Index** \| **Help** \| **Weka's home** | |  |
| **PREV CLASS**   **NEXT CLASS** | **FRAMES**    **NO FRAMES**     **All Classes** |  |
| SUMMARY: NESTED \| FIELD \| CONSTR \| METHOD | DETAIL: FIELD \| CONSTR \| METHOD |  |


---
